# Feasibility of an Individualized Exercise Training Program for Children with Hypertrophic Cardiomyopathy

**DOI:** 10.1007/s00246-025-03843-3

**Published:** 2025-04-15

**Authors:** Julianne Wanner, Danielle Burstein, Stephen Paridon, Maully Shah, Shannon O’Malley, Kimberly Y. Lin, Jonathan B. Edelson

**Affiliations:** 1 Division of Cardiology, The Children’S Hospital of Philadelphia, Philadelphia, PA USA; 2 Campbell University School of Osteopathic Medicine, Lillington, NC USA; 3 The University of Vermont Medical Center, Burlington, VT USA

**Keywords:** Hypertrophic cardiomyopathy, Pediatric, Exercise, Quality of life

## Abstract

Limited data exist to guide exercise participation in pediatric hypertrophic cardiomyopathy (HCM) patients. Exercise is traditionally restricted in this population, despite research in adults with HCM demonstrating exercise-associated cardiovascular and psychological benefit. This pilot study seeks to assesses the safety and feasibility of a moderate-intensity exercise program in children with HCM to guide future study design. Eight subjects ages 8–18 with HCM were recruited from the outpatient cardiology clinic at a large tertiary care referral center. All subjects were NYHA Class I (*n* = 4) or II (*n* = 4) and median septal wall thickness was 2.3 cm [IQR 1.4, 2.5]. The intervention included a 16-week exercise program, consisting of three 30-min aerobic and two strength sessions per week. A target heart rate zone was established using a baseline cardiopulmonary exercise test (CPET). A FitBit device was used to titrate activity to this zone during sessions. Self-reported adherence ranged from 1.25 to 96.25%. At baseline, cardiorespiratory fitness, assessed by CPET, and Health Related Quality of Life (HRQoL), assessed using two validated instruments, were reduced compared to peers. All subjects (*n* = 4) who completed the intervention improved in exercise performance and HRQoL measures. Ambulatory rhythm monitoring did not reveal any intervention-related arrhythmias, and there were no cardiac adverse events. This pilot study shows the promise of a potential benefit of an interventional exercise program in children with HCM. Future work should aim to improve retention, and more fully assess the safety and impact of exercise in pediatric HCM.

**Supplementary Information:**

The online version contains supplementary material available at 10.1007/s00246-025-03843-3.

## Background

### Introduction

Traditionally, the approach to physical activity in children with hypertrophic cardiomyopathy (HCM) has been restrictive, with concerns that the catecholamine surge that accompanies exercise could precipitate ventricular arrhythmia and potential sudden cardiac death (SCD), or that routine exercise could lead to pathologic remodeling and accelerate disease progression. Unfortunately, this restriction comes with a substantial cost; children with HCM are less active than their peers and have impaired levels of cardiovascular fitness compared to children without cardiac disease [1–3]. Furthermore, the prevalence of acquired cardiovascular risk factors, including obesity, hyperlipidemia, and hypertension [4] are unacceptably high in children and adolescents with HCM.

However, recent work has called this traditional practice of restriction into question. Population studies of children and young adults with HCM found that the majority of SCD events occurred during routine activities including rest or sleep, suggesting that vigorous physical activity may not be the inciting factor for SCD in many HCM patients [5, 6], and animal models have shown that exercise may have beneficial histologic effects by preventing or reversing fibrosis and myocyte disarray [7]. More recently, guidelines have changed, with the American Heart Association now acknowledging that the beneficial effects of exercise on health should be extended to patients with HCM [8]. These recommendations are based in part on studies showing the safety and efficacy of exercise for improving cardiovascular fitness in adults with HCM [9].

These encouraging findings noted, the safety and efficacy of exercise-related interventions in children with HCM likely differs from adults. It is critical that pediatric-specific investigations take place before changing pediatric exercise guidelines in this population. As an early step in this investigative pathway, we performed a pilot interventional exercise study in pediatric patients with HCM, with the goal of informing future trials aimed safely at increasing exercise capacity and physical activity in these children.

## Methods

### Study Design and Population

In this single-center prospective interventional pilot feasibility study, potential subjects between ages 8 and 18 with a diagnosis of HCM were recruited from an outpatient cardiomyopathy clinic at a large tertiary care children’s hospital. To meet the criteria for HCM and possible study inclusion, subjects were required to have the presence of unexplained left ventricular hypertrophy in any wall segment (either by echocardiogram or cardiac MRI) and to be thought to have HCM by their primary cardiologist. If the primary treating physician was still evaluating for HCM vs. another potential etiology (i.e. athlete’s heart, hypertension-associated hypertrophy) they were deemed ineligible. Additional inclusion criteria included computer and/or internet access to upload health data from activity monitors. Individuals with a family history of sudden cardiac death, non-sustained ventricular tachycardia, septal thickness > 3 cm, current or recommended implantable cardioverter-defibrillator (ICD), resting left ventricular outflow tract obstruction (LVOTO) gradient > 60mmhg, inability to complete cardiopulmonary exercise test (CPET) or ambulatory rhythm monitor, absence of regular atrial rhythm on baseline EKG, life expectancy < 12 months, or other comorbid congenital disease or associated syndrome were excluded from participating. If the patient or their parent/guardian were unable to complete study instruments due to cognitive disability or inability to answer English language questionnaires, they were excluded because the primary study endpoints depended on responses to study instruments. At the time of this study, some instruments were not available in other languages (Table 1).

**Table 1:** Inclusion/exclusion criteria

Inclusion criteria	Exclusion criteria
Age 8–18 years old	Family history of sudden cardiac death, personal history of non-sustained VT, unexplained syncope, abnormal blood pressure response to exercise, septal thickness > 3 cm, presence of an ICD or recommended ICD
Diagnosis of HCM, defined by presence of unexplained left ventricular hypertrophy > 14 mm in any wall segment	Resting LVOTO gradient > 60mmHG or under evaluation for septal reduction intervention
Agreement to participate in study protocol and willing/able to return for follow-up	Inability to complete a standard maximal CPET (as defined by RER > 1.1)
Access to computer and/or internet to upload health data from wearable activity monitor	NSVT on baseline exercise test or ambulatory rhythm monitor
	Absence of regular atrial rhythm on baseline EKG
	Unwillingness to refrain from high intensity exercise (competitive or organized sports, burse activity, heavy isometric exercise) for the duration of the study
	Pregnancy
	Life expectancy < 12 months
	Presence of other comorbid congenital heart disease and/or associated syndromes
	Parent/guardian or child/adolescent unable to answer forms due to inability to answer English language questionnaires
	Genetic syndrome or developmental delay which would make questionnaire and/or exercise test data uninterpretable

### Intervention Components

Subjects were provided with an individualized 16-week moderate-intensity exercise program consisting of at least 30 min of exercise per session, and at least five exercise sessions per week. Subjects were instructed to perform at least 3 days of aerobic exercise and 2 days of musculoskeletal training each week using resistance bands provided by the study. Sessions were not monitored in real-time, but heart rate data was visible by study team members after the exercise session on the provider dashboard. Exercise recommendations were individualized, utilizing available community resources (i.e. nearby parks, gyms, or home exercise equipment) and could be performed whenever best-suited subjects’ schedules. Each subject was given a FitBit device to allow them to titrate their activity to a target heart rate zone. Target heart rates were identified as the heart rate at the anaerobic threshold from a recent baseline CPET which typically occurs at 50–60% of maximal exercise intensity. The MyHeart CHOP application was added to the Fitbit device to simplify the target heart rate zones into colored zones. Weekly check-ins were performed by a study team member. Adherence was tracked using both a mobile health application (MyHeart CHOP) and self-reporting from subjects during weekly check-in calls.

### Study Measures

Subjects completed a CPET at baseline to identify a heart rate zone consistent with moderate-intensity exercise and assess cardiorespiratory fitness. Baseline echocardiograms were performed within 6 months prior to enrollment. A Mobile Cardiac Outpatient Telemetry Patch System was worn during the first 7 days of exercise training to assess for arrhythmias. Repeat CPET was performed at study conclusion.

Given the known associations of physical activity and emotional well-being, health-related quality of life (HRQoL) was assessed before and after the intervention using two validated instruments, the Pediatric Quality of Life Inventory (Peds-QL) and Pediatric Cardiac Quality of Life Index (PCQLI). Health-related quality of life as defined as “a multidomain concept that represents the subject’s general perception of the effect of illness and treatment on physical, psychological, and social aspects of life [10].” The Peds-QL assesses perceived HRQoL in patients with chronic conditions [11], while the PCQLI is a disease-specific instrument for measuring health-related quality of life in pediatric patients with congenital or acquired heart disease. In addition to a total score, the PCQLI includes subscales such as disease impact and psychosocial impact which measure the perceived impact of the disease on a child’s ability to function in different domains. Disease impact (DI) measures physical functioning whereas psychosocial impact (PI) measures psychological and social function. The maximum total score for the PCQLI is 100, and the maximum subscale score is 50 [12]. For both instruments, a higher score is indicative of a better perceived quality of life [11]. Both the PedsQL and PCQLI include parent proxy forms, measuring the parent or guardian’s perception of their child’s HRQoL, in addition to measuring the child’s perception of their own HRQoL.

Adherence to study intervention was calculated by dividing the total number of exercise sessions completed by the number of expected sessions (80) and converting to a percentage. Exercise sessions were tracked during weekly check-in calls via subject self-reporting, as well as through the mobile application. Both self-reported adherence and mobile application adherence were calculated for each subject.

### Covariates of Interest

Additional demographic and clinical data were abstracted from subjects’ medical record. Demographic data included age, sex, race and ethnicity. Clinical characteristics of interest included New York Heart Association (NYHA) class, history of arrhythmia, previous cardiac imaging findings, BMI, comorbid conditions, concomitant medications, and genotype status.

## Results

Over the course of 22 months, forty-one potential subjects were identified by the study team as potentially eligible. Of those, 11 were found to be ineligible during screening, with the primary reason being ICD placement or planned placement (*n* = 8). Potential subjects were approached sequentially at the time of their scheduled clinic visit, and 8 subjects ranging from age 8 to 18 were enrolled in the study.

As seen in Table 2, the study cohort comprised primarily males 75% (*n* = 6) and 2 subjects identified as Black race. All subjects were non-Hispanic or Latino and were New York Heart Association (NYHA) Class I (50%) or II (50%). Five of the eight subjects had previous clinically performed genetic testing. Of those, two subjects were genotype positive, one subject was genotype negative, and two subjects had variants of unknown significance. One subject had a history of both atrial and ventricular ectopy. On baseline echocardiogram, the median septal wall thickness was 2.3 cm and all subjects had normal systolic function. At the time of enrollment, 7 subjects had a previous cardiac MRI, with 71% (*n* = 5) having evidence of myocardial fibrosis by late gadolinium enhancement (LGE). One-half of the study cohort had a comorbid psychiatric diagnosis (attention-deficit/hyperactive disorder *n* = 2, mood disorder *n* = 1, anxiety disorder *n* = 1), and three subjects (36.5%) had a body mass index greater than 30 kg/m^2^. Five subjects were taking clinically prescribed cardiac medications during the study, including beta blockers (*n* = 3), calcium channel blockers (*n* = 1) and ACE inhibitors (*n* = 1).

**Table 2:** Demographics and clinical characteristics

Variable	*N* (%) or Median [IQR 25, IQR 75]
Age	16 [9.5,17]
Gender	
Male	6 (75%)
Female	2 (25%)
Race	
Black	2 (25%)
White	6 (75%)
Ethnicity	
Non-hispanic or latino	8 (100%)
NYHA class	
NYHA class I	4 (50%)
NYHA class II	4 (50%)
History of ventricular arrhythmia	1 (12.5%)
History of atrial arrhythmia	1 (12.5%)
Maximum septal wall thickness (cm)	2.3 [1.4, 2.5]
LV ejection fraction (%)	67.5 [62, 73.5]
LV shortening fraction (%)	48 [43.5, 50]
Presence of LGE on cardiac MRI (*n* = 7)	5 (71%)
Comorbid conditions	
Psychiatric diagnosis	4 (50%)
Obesity	3 (37.5%)
BMI	25.8 [18.75, 33.5]
Medications	
Beta blocker	3 (37.5%)
Calcium channel blocker	1 (12.5%)
ACE inhibitor	1 (12.5%)
LVOT peak gradient in subjects with obstruction (mmHg; *n* = 5)	17 [9, 51]
LVOT peak gradient all subjects (mmHg; *n* = 8)	9 [0, 30.5]
Genotype status	
Positive	2 (25%)
Negative	1 (12.5%)
Variant of unknown significance	2 (25%)
Genetic testing not performed	3 (37.5%)

*LV* left ventricle, *LGE* Late Gadolinium Enhancement, *BMI* Body Mass Index, *ACE* Angiotensin Converting Enzyme, *LVOT* Left Ventricular Outflow Track

On baseline CPET, subjects’ resting heart rate (HR) was 77 beats per minute (IQR 67, 85) with a maximum HR during exercise of 185 beats per minute. No subjects had an abnormal BP response to exercise. Cardiorespiratory fitness among this cohort was low, with a median maximal physical working capacity of 1.78 watts/kg (IQR 1.23, 2.46), and median maximal oxygen consumption of 27.3 ml/kg/min (IQR 23.9, 35.2) and 70% (IQR 59, 76) of subjects’ predicted value [13]. Two subjects had ectopy at peak exercise, including one who had PACs and a monomorphic wide complex couplet, and another with PVC’s of at least two morphologies. There were no symptoms reported by subjects during CPET, and there were no subjects with exercise-associated ischemic changes on ECG (Table 3).

**Table 3:** Baseline EST

Baseline exercise stress test	N (%) or median [IQR25, IQR 75]
HR Rest	77 [67,85]
HR Anaerobic threshold	147 [119, 156]
Max HR	185 [149, 202]
Systolic BP at rest (mmHg)	118 [110, 123]
Maximum systolic BP (mmHg)	152 [135, 156]
Resting systolic BP > 130 mmHg	0 (0%)
Abnormal BP response to EST	0 (0%)
Anaerobic threshold PWC (watts)	86 [31, 102]
Anaerobic threshold PWC (watts/kg)	0.97 [0.37, 1.54]
Maximal PWC (watts)	143 [70, 203]
Maximal PWC (watts/kg)	1.78 [1.23, 2.46]
Anaerobic threshold O2 Con. (L/min)	1.41 [0.81, 1.72]
Anaerobic threshold O2 Con. (ml/kg/min)	19.2 [15.5, 22.7]
Max O2 consumption L/min	1.82 [1.21, 2.59]
Max O2 consumption (% of predicted value)^1^	70 [59, 76]
Max O2 consumption ml/kg/min	27.3 [23.85, 35.15]
Sinus rhythm at rest	8 (100%)
Ectopy	2 (25%)
Ischemic changes (*n* = 4)	0
Symptoms	0 (0%)

*PWC* Peak Working Capacity

^1^Max O2 consumption (% of predicted value) was calculated using reference values established by Burstein et al. (2021) “Normative Values for Cardiopulmonary Exercise Stress Testing Using Ramp Cycle Ergometry in Children and Adolescents” [13]

At baseline, child reported PedsQL (median 67.2) and PCQLI (median 63.2) scores were abnormally low, indicating poor perceived quality of life in this cohort. Two subjects had PedsQL scores less than 50 and one had a PCQLI score less than 50. Subscale scores, measuring impact of disease on physical functioning (DI) and psychological/social functioning (PI), were also low with PCQLI DI and PCQLI PI median scores of 31.91 and 31.95, respectively, out of a total possible subscale score of 50. Parent proxy PedsQL median scores were higher than subject-reported scores, with a median of 78.33. PCQLI total and subscale scores were similar among parents and subjects. There were no PCQLI or PEDSQL total parent-reported scores less than 50. Additional information pertaining to parent and child quality of life measures at baseline can be found in supplemental Table 1.

Half of the enrolled subjects completed the study by returning for all study completion assessments. One subject withdrew from the study after week two, stating they did not feel as though they could keep up with the demands of the program given competing responsibilities, and two subjects were lost to follow up. One subject did not return for the final study completion visit, and thus could not be included in the assessment of outcomes measures. Subjects’ self-reported exercise adherence ranged from 1/80 (1.25%) to 77/80 (96.25%) completed sessions and was higher than exercise adherence calculated using the exercise tracking app in 7/8 subjects. The number of weekly check-in calls (maximum 16) completed by each subject ranged from 1 to 16, with a median of 10/16 completed check-ins. Qualitative responses during weekly check-in calls revealed a variety of motivating factors for exercise, including parental encouragement, intrinsic motivation, and enjoying exercising with others (Table 4).

**Table 4:** Adherence

Subject ID	Self-reported adherence (%)	App reported adherence	Number of weekly check-ins completed (16 maximum)	Qualitatively reported motivating factors
1	77/80	80/80	16/16	“self motivated, want to get more endurance, want to get stronger, exercise with friends”
2	31/80	17/80	7/16	
3	7/80	3/80	3/16	
4	28/80	4/80	8/16	“motivated by mom, swimming is fun, motivated by weather”
5	68/80	53/80	16/16	“motivated by mom”
6	1/80		1/16	Withdrew at week 2
7	51/80	40/80	14/16	“motivated to lose weight/get in shape”
8	50/80	21/80	12/16	“motivated by mom and dad, motivated by playing with friends”

Three subjects reported a total of 4 adverse events, ranging from mild to moderate severity, and none of which were thought to be cardiac in etiology or intervention related. Detailed explanations of adverse events can be found in Table 5.

**Table 5:** Adverse events

Subject ID	Adverse event	Description
1	Chest pain	Chest pain likely musculoskeletal, as chronicity and severity were atypical of cardiogenic chest pain ECG unchanged from baseline and troponin negative
4	Coughing	Consistent with known history of seasonal allergies
8	Chest pain	Chest pain while resting at school (reading a book) Pain rated 9/10 Heart rate 122 at time of symptoms
8	Chest pain	Similar in nature to prior episode. Resolved without intervention

Given the small sample size, it is difficult to comment on the efficacy of the intervention. However, it is interesting to note that maximal working capacity increased from baseline by greater than 5 watts in the three returning subjects with exercise stress test data, and that all returning subjects showed at least one improved subject-reported quality of life measure from baseline to follow up. The greatest change in scores was seen in the PCQLI Disease Impact subscale, a measurement of perceived physical functioning. Additional detailed data pertaining to outcomes in returning subjects can be found in supplemental Tables 2, 3 and 4.

## Discussion

The medical community’s perspective towards physical activity in children with HCM is rapidly changing, but there remain limited studies in children to guide programs that are both safe and effective. As a step in that investigative pathway, we have completed a pilot interventional exercise program in pediatric HCM that has several important findings. First, in this carefully selected, small cohort, there were no cardiac-related adverse events. Second, preliminary estimates of efficacy showed improvements in measures of both exercise performance and quality of life. Finally, this experience demonstrates that developing strategies to promote adherence is necessary so that the benefits of an interventional exercise program can be widely delivered.

This heterogeneous cohort of children with HCM included subjects with a genotype-positive status, and with clinical manifestations that included a history of arrhythmia and myocardial fibrosis on cardiac MRI. We included several measures to ensure the safety of participating subjects, including placement of a 7-day rhythm monitor at program onset, review of safe exercise techniques with an exercise physiologist, and weekly check-ins to assess for any concerning signs or symptoms. As in other studies [14], we used a wearable device to help guide unmonitored exercise sessions, with subjects asked to not exceed a heart rate that correlated with more than moderate-intensity exercise. Fortunately, there were no study-related adverse events in these subjects, which suggests that there is a population of pediatric patients with HCM that may be able to safely participate in exercise-related interventions, with appropriate patient selection, education, and supervision in place.

This study demonstrated several interesting findings regarding the potential efficacy of this type of intervention. Despite abnormally low exercise performance at baseline, many exercise performance measures improved in subjects who returned for follow-up visits, with greater improvement observed in subjects with higher adherence to the exercise intervention. These promising results are similar to those seen in adult studies [15] and suggest that interventional exercise programs have the potential to be efficacious in children with HCM. This is important for several reasons. Exercise capacity is an important end its own right, as it means these children may be more likely to participate and enjoy the physical activities of childhood. Perhaps more importantly, increasing exercise capacity in these children is potentially a pathway towards improving HCM-specific cardiac health and reducing the acquired cardiovascular risk factors that come with inactivity, including obesity, hyperlipidemia, hypertension, and elevated blood glucose levels.

At baseline, this study cohort demonstrated poor health-related quality of life, and a high prevalence of comorbid psychiatric diagnoses, a finding that mirrors other studies showing impaired emotional well-being in children with HCM [16]. There are many reasons that children with HCM may have impaired HRQOL, including coping with the symptoms of their disease, the emotional burden of having a life-long illness, and the need for some to stop participating in preferred activities. While this study was not designed to assess the etiology of these findings, the results suggest a clear need for attention and intervention in the mental health of this patient population. Notably, those subjects who completed the exercise intervention had improvements in their measures of HRQOL. We suspect that this relationship is likely bidirectional rather than causal, in that children with improved HRQOL may be more able to engage in a long-term exercise intervention that requires motivation and discipline. However, given the well-established benefits of exercise on mental health [17, 18], we should not discount the possibility that an exercise intervention could improve deficiencies in emotional well-being, and increased attention to this phenomenon is warranted in future studies.

It is important to recognize that only 4 of the 8 subjects who enrolled in the intervention completed the study, with some subjects dropping out due to competing responsibilities and others lost to follow-up. Behavioral interventions such as this are traditionally challenging in terms of retention, however, there were several lessons learned that can potentially inform future efforts. During weekly check-ins, subjects noted the importance of parental encouragement and social incentives. This raises the possibility that family based and/or group-based interventions may be effective. Additionally, subjects often reported challenges with the mobile application and wearable devices. While the use of this technology can provide valuable heart rate information to guide the intervention, its malfunction presents challenges for both subjects and researchers. This may be an important consideration for future study design. We also suspect that including non-academic invested parties’ input in the design of future interventions is likely to lead to more effective and broadly applicable interventions. Finally, it is important to recognize that suboptimal adherence could be a limitation to enjoying the benefits of an exercise intervention program. Prior research in children with congenital heart disease has demonstrated that expanded inclusion criteria and monitored exercise sessions may encourage participation [19]. Future iterations should build on these findings, with attention to modifications aimed at improving adherence and retention.

## Limitations

There are several limitations to this study which should be acknowledged. The small sample size precludes us from commenting on the generalizability of these findings, and there may have been inadvertent selection bias, with children more likely to adhere and benefit from this type of intervention more likely to enroll. Due to technological challenges encountered with the MyHeart CHOP exercise tracking application, we relied heavily on assessment of adherence using subject-reported data, which may have introduced reporting bias. As we do not have longer-term follow-up, we are unable to comment on the sustainability of the observed changes.

## Conclusion

This pilot study is an important step in developing interventions that develop safe and effective physical activity patterns in children with HCM. This small cohort of pediatric patients experienced no adverse events and showed improvements in measures of exercise capacity and quality of life. While these findings emphasize the potential utility of physical activity-related interventions in this population, future iterations that include participant input in the design of the intervention are needed to increase adherence, sustainability, and generalizability.

## Supplementary Information

Below is the link to the electronic supplementary material.

Supplementary file1 (DOCX 18 KB)

## Data Availability

Deidentified data will be shared upon request and after execution of a data use agreement.
