# Supplementary material for: Feasibility of an Individualized Exercise Training Program for Children with Hypertrophic Cardiomyopathy
Source: Pediatr Cardiol. 2025 Apr 15;47(2):795–802. doi: 10.1007/s00246-025-03843-3 (PMC12855382; doi:10.1007/s00246-025-03843-3)
Supplement: Supplementary file 1 — Supplementary file1 (DOCX 18 KB) [file 246_2025_3843_MOESM1_ESM.docx]

**Supplemental Materials**

**Supplemental Table 1: Cohort Quality of Life at Baseline**

| Questionnaire | Child Median  [IQR25, IQR75]  n = 8 | Parent Proxy Median [IQR25, IQR75]  n = 7 |
| --- | --- | --- |
| Baseline PedsQL | 67.2 [49.17, 73.99] | 78.57 [57.14, 86.67] |
| Baseline PCQLI Total | 63.17 [57.29, 71.85] | 60.42 [56.99, 73.22] |
| Baseline PCQLI DI | 31.91 [28.13, 34.84] | 31.25 [25.74, 33.93] |
| Baseline PCQLI PI | 31.95 [28.48, 34.9] | 31.25 [29.17, 40.28] |

**Supplemental Table 2: Exercise Stress Test Changes**

| Subject ID | 1 | 2 | 5 |
| --- | --- | --- | --- |
| Resting HR (pre) | 84 | 55 | 86 |
| Resting HR (post) | 78 | 63 | 84 |
| Resting BP (pre) | 118/66 | 114/52 | 122/75 |
| Resting BP (post) | 124/54 | 106/50 | 108/70 |
| PWC at AT (pre) | 105 watts  1.65 watts/kg | 102 watts  1.54 watts/kg | 25 watts  0.37 watts/kg |
| PWC at AT (post) | 120 watts  1.87 watts/kg | 92 watts  1.36 watts/kg | 42 watts  0.58 watts/kg |
| PWC at Max (pre) | 203 watts  3.2 watts/kg | 163 watts  2.46 watts/kg | 63 watts  0.94 watts/kg |
| PWC at Max (post) | 235 watts  3.65 watts/kg | 169 watts  2.50 watts/kg | 80 watts  1.11 watts/kg |
| Max VO2 (pre) | 3.27 L/min  51.5 ml/kg/min | 1.98 L/min  29.9 ml/kg/min | 1.66 L/min  24.7 ml/kg/min |
| Max VO2 (post) | 2.53 L/min  39.2 ml/kg/min | 2.29 L/min  33.8 ml/kg/min | 1.73 L/min  24.3 ml/kg/min |

**Supplemental Table 3: Child Quality of Life**

| Subject | Baseline  PedsQL | Follow up Peds QL | Baseline  PCQLI Total | F/u PCQLI Total | Baseline  PCQLI DI | F/u PCQLI DI | Baseline PCQLI PI | F/u PCQLI  PI |
| --- | --- | --- | --- | --- | --- | --- | --- | --- |
| 1 | 93.33 | 95 | 75.92 | 85.54 | 35.29 | 39.71 | 40.63 | 45.83 |
| 2 | 68.33 | 70 | 67.78 | 66.85 | 34.35 | 34.56 | 33.33 | 32.29 |
| 3 | 46.67 |  | 48.1 |  | 22.06 |  | 26.04 |  |
| 4 | 33.33 |  | 58.63 |  | 29.46 |  | 29.17 |  |
| 5 | 69.64 | 68.33 | 56.35 | 66.77 | 28.57 | 34.82 | 27.78 | 31.94 |
| 6 | 66.07 |  | 67.71 |  | 34.38 |  | 33.33 |  |
| 7 | 78.33 |  | 76.16 |  | 39.71 |  | 36.46 |  |
| 8 | 51.67 | 64.29 | 58.23 | 75.20 | 27.68 | 32.14 | 30.56 | 43.06 |

|  | Score increase >5 points |
| --- | --- |

**Supplemental Table 4: Parent Proxy Quality of Life**

| Subject | Baseline PedsQL | Follow up PedsQL | Baseline  PCQLI Total | F/u PCQLI Total | Baseline PCQLI DI | F/u PCQLI DI | Baseline PCQLI PI | F/u PCQLI PI |
| --- | --- | --- | --- | --- | --- | --- | --- | --- |
| 1 | 96.67 | 98.33 | 72.98 | 88.48 | 32.35 | 42.65 | 40.63 | 45.83 |
| 2 | 57.14 | 56.67 | 56.99 | 63.17 | 25.74 | 30.88 | 31.25 | 32.29 |
| 3 |  |  |  |  |  |  |  |  |
| 4 | 53.33 |  | 51.19 |  | 30.36 |  | 20.83 |  |
| 5 | 68.33 | 70 | 60.42 | 59.13 | 31.25 | 28.57 | 29.17 | 30.56 |
| 6 | 78.57 |  | 56.99 |  | 25.74 |  | 31.25 |  |
| 7 | 86.67 |  | 73.22 |  | 36.76 |  | 36.46 |  |
| 8 | 78.85 | 73.33 |  |  |  |  |  |  |

|  | Score increase >5 points |
| --- | --- |
|  | Score decrease >5 points |

**Supplemental Table 5: Change in Quality of Life**

| Subject | Change in Subject Peds QL | Change in Subject PCQLI total | Change in Subject PCQLI DI | Change in Subject PCQLI PI | Change in Parent Peds QL | Change in Parent PCQLI total | Change in Parent PCQLI DI | Change in Parent PCQLI PI |
| --- | --- | --- | --- | --- | --- | --- | --- | --- |
| 1 | 1.67 | 9.62 | 4.42 | 5.2 | 1.66 | 15.5 | 10.3 | 5.2 |
| 2 | 1.67 | -0.93 | 0.21 | -1.04 | -0.47 | 6.18 | 5.14 | 1.04 |
| 5 | -1.31 | 10.42 | 6.25 | 4.16 | 1.67 | -1.29 | -2.68 | 1.39 |
| 8 | 12.62 | 16.97 | 4.46 | 12.5 | -5.52 |  |  |  |

|  | Score increase >5 points |
| --- | --- |
|  | Score decrease >5 points |
